# Genetic alterations and their therapeutic implications in epithelial ovarian cancer

**DOI:** 10.1186/s12885-021-08233-5

**Published:** 2021-05-04

**Authors:** Nina Lapke, Chien-Hung Chen, Ting-Chang Chang, Angel Chao, Yen-Jung Lu, Chyong-Huey Lai, Kien Thiam Tan, Hua-Chien Chen, Hsiao-Yun Lu, Shu-Jen Chen

**Affiliations:** 1 ACT Genomics, Co. Ltd., 3F., No.345, Xinhu 2nd Rd., Neihu Dist, Taipei City, 114 Taiwan; 2 ACT Genomics, Co. Ltd., Units 803 – 807, 8F, Building 15W, No.15 Science Park West Avenue, Hong Kong Science Park, Pak Shek Kok. NT, Hong Kong, Hong Kong; 3 Department of Obstetrics and Gynecology, Chang Gung Memorial Hospital and Chang Gung University, Linkou Medical Center, 5 Fushin St., Guishan District, Taoyuan, 333 Taiwan; 4 Gynecologic Cancer Research Center, Chang Gung Memorial Hospital, 5 Fushin St., Guishan District, Taoyuan, 333 Taiwan

**Keywords:** Ovarian cancer, Histology, High-grade serous carcinomas, Endometrioid carcinomas, Clear cell carcinomas, Next-generation sequencing, Genetic alterations, Signaling pathway, Targeted therapy

## Abstract

**Background:**

Genetic alterations for epithelial ovarian cancer are insufficiently characterized. Previous studies are limited regarding included histologies, gene numbers, copy number variant (CNV) detection, and interpretation of pathway alteration patterns of individual patients.

**Methods:**

We sequenced 410 genes to analyze mutations and CNV of 82 ovarian carcinomas, including high-grade serous (*n* = 37), endometrioid (*n* = 22) and clear cell (*n* = 23) histologies. Eligibility for targeted therapy was determined for each patient by a pathway-based approach. The analysis covered DNA repair, receptor tyrosine kinase, PI3K/AKT/MTOR, RAS/MAPK, cell cycle, and hedgehog pathways, and included 14 drug targets.

**Results:**

Postulated PARP, MTOR, and CDK4/6 inhibition sensitivity were most common. *BRCA1/2* alterations, *PTEN* loss, and gain of *PIK3CA* and *CCND1* were characteristic for high-grade serous carcinomas. Mutations of *ARID1A*, *PIK3CA*, and *KRAS*, and *ERBB2* gain were enriched in the other histologies. *PTEN* mutations and high tumor mutational burden were characteristic for endometrioid carcinomas. Drug target downstream alterations impaired actionability in all histologies, and many alterations would not have been discovered by key gene mutational analysis. Individual patients often had more than one actionable drug target.

**Conclusions:**

Genetic alterations in ovarian carcinomas are complex and differ among histologies. Our results aid the personalization of therapy and biomarker analysis for clinical studies, and indicate a high potential for combinations of targeted therapies.

**Supplementary Information:**

The online version contains supplementary material available at 10.1186/s12885-021-08233-5.

## Background

Ovarian cancer accounts for about 300,000 new diagnoses and 185,000 deaths annually, being both the eighth most common and deadly cancer in women worldwide [1]. In the United States, it is ranked the fifth deadliest cancer type in females, and therefore remains a major health concern despite its decreasing incidence and death rates [2]. Ovarian cancer is a heterogeneous disease, with serous, endometrioid and clear cell histologies being common. The subtype-specific prevalence depends on the geographic origin, being about 45–60%, 10–15 and 5% in Western countries, and 23–34%, 14–18% and 14–21% in Asian countries, respectively [3].

Histological subtypes may respond differently to therapy, e.g. chemotherapy [4, 5], and genetic differences between subtypes may contribute to this phenomenon. The value of tumor genetic testing for targeted therapies is demonstrated by the EMA and USFDA approval of PARP (Poly (ADP-ribose) polymerase) inhibitors, PARPi, for patients with *BRCA1/2* mutations. Recent research provides further evidence that the use of biomarkers in clinical trials, especially of genomic biomarkers, is associated with better outcomes [6, 7]. Genomic biomarkers could therefore identify patients who may benefit from drugs that have yielded disappointing results in patients who were unselected or could not be sufficiently stratified by other markers [8, 9]. Such an approach could also prevent treatment-associated toxicities in patients unlikely to respond. Genetic patient selection was demonstrated to be a promising approach [10–12], although studies are mostly limited to certain key genes without considering whole signaling pathways.

It is known that different histological subtypes of ovarian cancer differ in their tumor genetic alterations, indicating that these patients may be better suited for therapies with different targeted agents [13–19], especially for the treatment of recurrence. However, many published studies focus on an analysis of isolated, commonly mutated genes [15–18], analyze limited exonic regions for included genes [14, 17, 18], and do not cover copy number variations (CNV) for any or most analyzed genes [14, 15, 18, 19]. Although research investigating CNV has resulted in the generation of chromosomal CNV profiles for different histological subtypes [20], information about co-occurring mutations and direct therapeutic implications remains limited. Studies analyzing genetic alterations more comprehensively have resulted in an improved estimation of the frequency of expected alterations in some cancer signaling pathways [13, 21, 22], the most comprehensive analysis being the TCGA study on high-grade serous carcinomas [13]. However, results are still limited regarding analyzed histological subtypes and signaling pathways. Studies further lack an interpretation of signaling pathway alteration patterns for individual patients to estimate the actionability of genes that are targets for therapeutic agents.

To enable an improved estimation of the proportion of ovarian cancer patients with different histological subtypes who are eligible for targeted therapies, we designed the present study. A hallmark of our study is the evaluation of gene actionability by a pathway-based approach and investigation of intra- and inter-pathway alteration patterns from individual patients. This approach allowed the identification of drug-sensitizing alterations, including those of less commonly altered pathway genes. In patients with sensitizing alterations, it further enabled the detection of potentially resistance-mediating downstream alterations. The analysis of concurrent alterations allowed the identification of options for a combination of targeted therapies. Our analysis was based on a comprehensive analysis of mutations and CNV to determine gene actionability, and the prediction of the impact of detected mutations by OncoKB or ACMG/AMP guidelines. It further included the comparison of genetic alteration frequencies and types in single genes of interest.

We analyze genetic alterations in tumor samples from 82 ovarian cancer patients, including patients with high-grade serous (*n* = 37), endometrioid (*n* = 22) and clear cell carcinomas (*n* = 23). Next-generation sequencing of more than 400 cancer-related genes was performed, and analyzed regions included all coding exons as well as exon-intron boundaries. Identified variants were single nucleotide variants, small insertions and deletions and CNV. The incentive of the study was to identify targeted agents that could be an option for therapy if recurrence occurs. For this purpose, we analyzed tumor genetic alterations with potential therapeutic implications for targeted therapies and immunotherapy.

## Methods

### Patients and samples

We retrospectively reviewed the medical records of women with epithelial ovarian cancer (Stage I-IV) who received primary surgical treatment at the Chang Gung Memorial Hospital, Linkou Medical Center (Taoyuan, Taiwan) between 2000 and 2005. Two expert pathologists independently reviewed all slides to avoid diagnostic inaccuracies regarding histology.

Samples from 85 patients were originally included in the study. Three samples with less than 20% of tumor purity, or for which tumor purity could not be determined, were excluded. The final study cohort consisted of 37 patients with high-grade serous, 22 patients with endometrioid, and 23 patients with clear cell carcinomas.

### Gene sequencing

Four hundred ten cancer-related genes were analyzed using two NGS cancer panels. The first panel used the Ion AmpliSeq Comprehensive Cancer Panel (Life Technologies), including 409 genes relevant for cancer research and therapy. Experimental procedures were as previously described [23], or otherwise specified. In brief, DNA was isolated from formalin-fixed paraffin-embedded (FFPE) tumor samples, amplified, and subsequently sequenced on an Ion proton sequencer. Of the 409 included genes, 408 genes were analyzed (the gene *PDE4DIP* was not included due to the high number of pseudogenes). The second panel included *BRCA1/2*. Similarly, tumor samples were FFPE specimens, with DNA isolation and sequencing procedures being as previously published [24]. Identified variants represent a mixture of germline and somatic variants. All genes included in the study are listed in Additional file 1.

The mean sequencing depth for the comprehensive cancer panel and the *BRCA1/2* panel was >1000x and > 6000x, and the mean uniformity was 90 and 91%, respectively. Detailed information for each case is listed in Additional file 2.

### Genetic alteration analysis

To determine genetic alterations, all covered coding exons and exon-intron boundaries included in the used panel were analyzed. Identified variants were single nucleotide variants, small insertions and deletions, which included both protein coding and splice-site variants, and CNV. The human genome sequence hg19 served as a reference genome, and alignment/base calling and variant calling were performed with the Torrent Suite Server version 5.0 and Torrent Suite Variant Caller plug-in, version 5.0, respectively. For analysis, the Ion Torrent default pipeline and default parameters were used.

Only mutational variants that had frequencies of at least 10% in a patient’s tumor sample and did not meet the criteria for polymorphisms were included in the further analysis. Polymorphisms were variants that were 1) included in the 1000 Genomes Project (populations: global, ASN, AFR, AMR, EUR, AA, EA), Genome Aggregation Database (gnomAD, populations: total, EAS, AFR, AMR, ASJ, FIN, NFE, OTH) or Exome Aggregation Consortium (ExAC, populations: total, Adj, AFR, AMR, EAS, FIN, NFE, OTH, SAS), and had an allele frequency ≥ 0.5%, or 2) detected in 24 peripheral blood mononuclear cell (PBMC) in-house samples from healthy Taiwanese volunteers.

CNV were analyzed using ONCOCNV (https://github.com/BoevaLab/ONCOCNV) [25]. The diploid genome baseline was established using our 24 in-house PBMC samples from healthy volunteers (ratio male: female = 1:1) as a reference. The ADTEx tool [26] was used to correct baseline shifts based on SNP information and estimate tumor purity. Copy number gain was defined as an observed copy number ≥ 4, whereas copy number loss was defined as an observed copy number ≤ 1.

Regarding *BRCA1/2* testing, we have previously reported variants, including their germline or somatic variant origin, in a study cohort overlapping with the present cohort [24]. As for the comprehensive gene panel, only *BRCA1/2* variants with a frequency of at least 10% in tumor samples were included in the current analysis.

### Tumor mutational burden

To calculate the tumor mutational burden (TMB) for individual patients, the number of detected mutations was divided by the number of analyzed base pairs (1.247752 Mb).

### Determination of gene actionability

The determination of gene actionability was based on genetic alterations in the respective pathways [27]. For our analysis, we considered pathways, drug targets or important pathway genes for which previous studies with a corresponding drug have been performed in ovarian cancer and for which the literature indicates that alterations in such genes can influence therapy outcome. Previous treatment efficacy in ovarian cancer treatment was not required for inclusion since it may have been related to a lack of predictive biomarkers. In total, we analyzed six pathways, 14 drug targets, and considered 54 genes. Pathways were 1) DNA repair, drug target PARP, included genes *ARID1A, ATM, ATR, BAP1, BLM, BRCA1, BRCA2, CHEK1, CHEK2, ERCC1, MLH1, MRE11A, PALB2, PTEN*, and *RAD50* [13, 28–37], 2) RTK, drug targets and considered genes *EGFR, ERBB2, KDR, IGF1R, MET,* and *RET* [9, 38–44], 3) PI3K/AKT/MTOR, drug targets PIK3CA, AKT1, and MTOR, considered genes *AKT1, FLCN, FBXW7, NF2, PIK3CA, PTEN, STK11, TSC1, TSC2*, and *MTOR* (additional genes: *AKT2, AKT3, PIK3CB, PIK3CD*, and *PIK3CG*) [10, 11, 13, 17, 45–56] 4) RAS/MAPK, drug targets KRAS, and MEK1/2, considered genes *KRAS, NF1, NF2, MAPK1, MAP 2 K1* and *MAP 2 K2* (additional genes: *BRAF, HRAS,* and *NRAS*) [10, 13, 55–58], 5) cell cycle, drug targets CDK4/6, considered genes *CCND1, CCNE1, CDKN2A, CDKN2B, CDK4, CDK6*, and *RB1* [13, 59–62], and 6) hedgehog, drug target SMO, considered genes *PTCH1, SMO, STK36*, and *SUFU* [8, 63, 64].

For analysis, oncogenes were defined as cancer growth signaling pathway activating genes. Tumor suppressor genes were defined as cancer growth signaling pathway inactivating genes, or genes involved in DNA repair. The following alterations were included in the therapeutic implication analysis: 1) oncogene gain, 2) tumor suppressor gene loss or truncating/splice variants, 3) variants classified “oncogenic” or “likely oncogenic” by OncoKB, and 4) *BRCA1/2* variants classified as “pathogenic” or “likely pathogenic” by the ACMG/AMP guidelines. All other variants were considered as VUS.

Gene actionability for the cancer growth pathways PI3K/AKT/MTOR, RAS/MAPK, cell cycle, and hedgehog was determined based on the hypothesis that activating alterations upstream of or on the drug target lead to gene actionability, whereas pathway activating gene alterations downstream of the drug target impair gene actionability. *PTEN* alterations were not considered for the determination of PIK3CA actionability.

### Statistical analysis and tools

Statistical comparisons between patient proportions of different histological subtypes were performed by Chi-Square Test. All statistical analyses were performed using GraphPad Prism (v. 6.0; GraphPad Inc.).

## Results

Eighty-five tumor samples were subjected to next-generation sequencing of more than 400 cancer-related genes to identify therapeutically relevant genetic alterations. A summary of the study design is provided in Additional file 3. The samples of three patients did not meet the study criteria for tumor purity. The final cohort therefore consisted of 82 patients, including 37 patients with high-grade serous, 22 patients with endometrioid, and 23 patients with clear cell carcinomas. Three of those patients had samples with tumor purities between 20 and 30% (high-grade serous, B00250, and clear cell, B00285 and B00287), which could impact the detection of CNV, especially for gene loss. Patient characteristics are provided in Additional file 4.

A complete list of all protein coding and splice mutations observed in our cohort is provided in Additional file 5. When evaluating therapeutic implications, all mutations and copy number alterations detected in genes of investigated pathways were considered as described in the Methods section. Relevant copy number variants, as well as gene mutations - and information about their OncoKB or ACMG/AMP classification, if applicable - are listed in Additional file 6. In the following description of our results, the terms “variant”, “mutation”, and “alteration” will not extend to variants of unknown significance (VUS), unless otherwise specified.

### Actionability of DNA repair-related alterations and tumor mutational burden

**Fig. 1:**
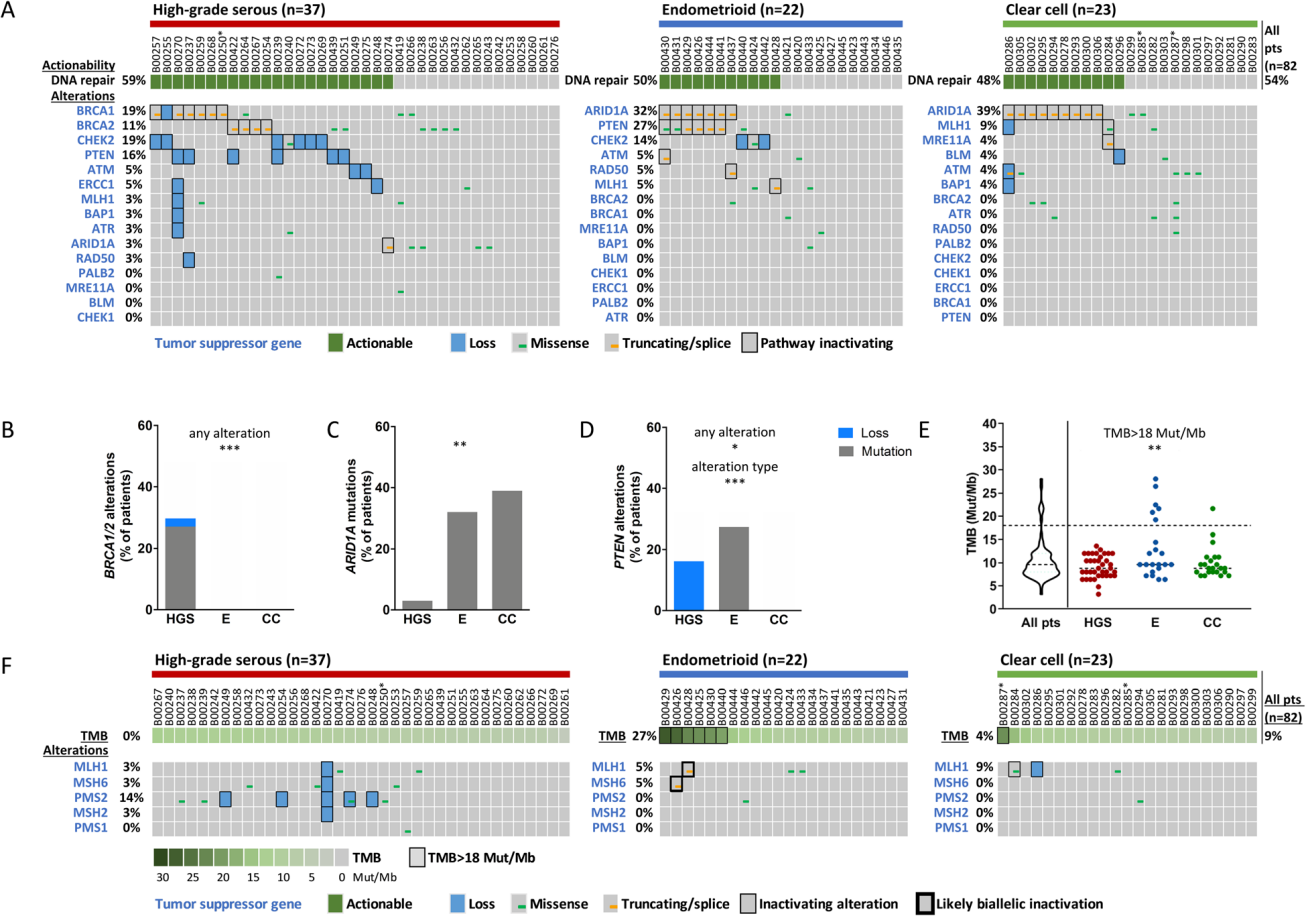
Alterations of DNA repair-related genes, resulting actionability and tumor mutational burden (TMB). Genetic alterations in DNA repair-related genes and their postulated actionability for PARP inhibition are depicted in oncoprint plots (**a**). The differential distribution of genetic alterations for *BRCA1/2* (**b**), *ARID1A* (**c**) and *PTEN* (**d**) between histological subtypes is shown in bar charts. The TMB distribution in the study cohort is depicted (**e**). Patients of the three histological subtypes were subdivided into those with a TMB > 18 Mut/Mb and < 18 Mut/Mb. All statistical analyses were performed by the Chi-Square test (see Additional file 7). Statistical significance is displayed as *P < 0.05, **P < 0.01, and ***P < 0.001. Genetic alterations in mismatch repair genes are depicted in oncoprint plots, together with the TMB (**f**). Histological subtypes are abbreviated as HGS = high-grade serous, E = endometrioid and CC = clear cell. In oncoprint plots, * indicates a sample with low (20–30%) tumor purity

Alterations in DNA repair-related genes that could be associated with PARPi sensitivity are depicted in Fig. 1a. DNA repair pathway-related actionability was postulated for 59, 50 and 48% of patients with high-grade serous, endometrioid and clear cell carcinomas, respectively.

For patients with high-grade serous carcinomas, the therapeutically well characterized genes *BRCA1/2* harbored variants in half of patients with DNA repair pathway actionability (30%, *n* = 11). However, *BRCA1/2* did not harbor alterations in any other histological subtype, and this differential distribution reached statistical significance (*P* < 0.001, Fig. 1b and Additional file 7).

In the majority of patients with endometrioid and clear cell carcinomas and potential actionability, *ARID1A* truncating or splice mutations were observed (7/11 and 9/11, respectively), whereas only one high-grade serous carcinoma patient harbored such a mutation (*P* = 0.001, Fig. 1c and Additional file 7).

*PTEN* had a unique alteration profile among histological subtypes. Inactivation of *PTEN* occurred only by copy number loss in high-grade serous carcinomas and by mutations in endometrioid carcinomas, while *PTEN* was not altered in clear cell carcinomas, and the differential distribution between histological subtypes was statistically significant (*P* = 0.033 for the detection of any *PTEN* alteration and *P* < 0.001 for different alteration types, Fig. 1d and Additional file 7).

A high tumor mutational burden (TMB) is associated with better outcome of immunotherapy. The median TMB in our cohort was 9.6 Mut/Mb, and the TMB was below 18 Mut/Mb for the majority of the cohort (91%, TMB range: 3.2–16.0 Mut/Mb, Fig. 1e). However, there were patients with a TMB > 18 Mut/Mb that differed from the low TMB observed in most patients (9%, TMB range: 19.2–28.1 Mut/Mb). A TMB > 18 Mut/Mb was more often observed in patients with endometrioid carcinomas (27%), compared to patients with high-grade serous and clear cell carcinomas (0 and 4%, respectively, *P* = 0.001, Fig. 1e and Additional file 7). Among patients with a high TMB, two patients with endometrioid carcinomas harbored each two variants in the DNA mismatch repair genes *MLH1* and *MSH6*, which is consistent with biallelic inactivation (Fig. 1f and Additional file 6).

### Gene actionability of receptor tyrosine kinases, the PI3K/AKT/MTOR and RAS/MAPK pathway

**Fig. 2:**
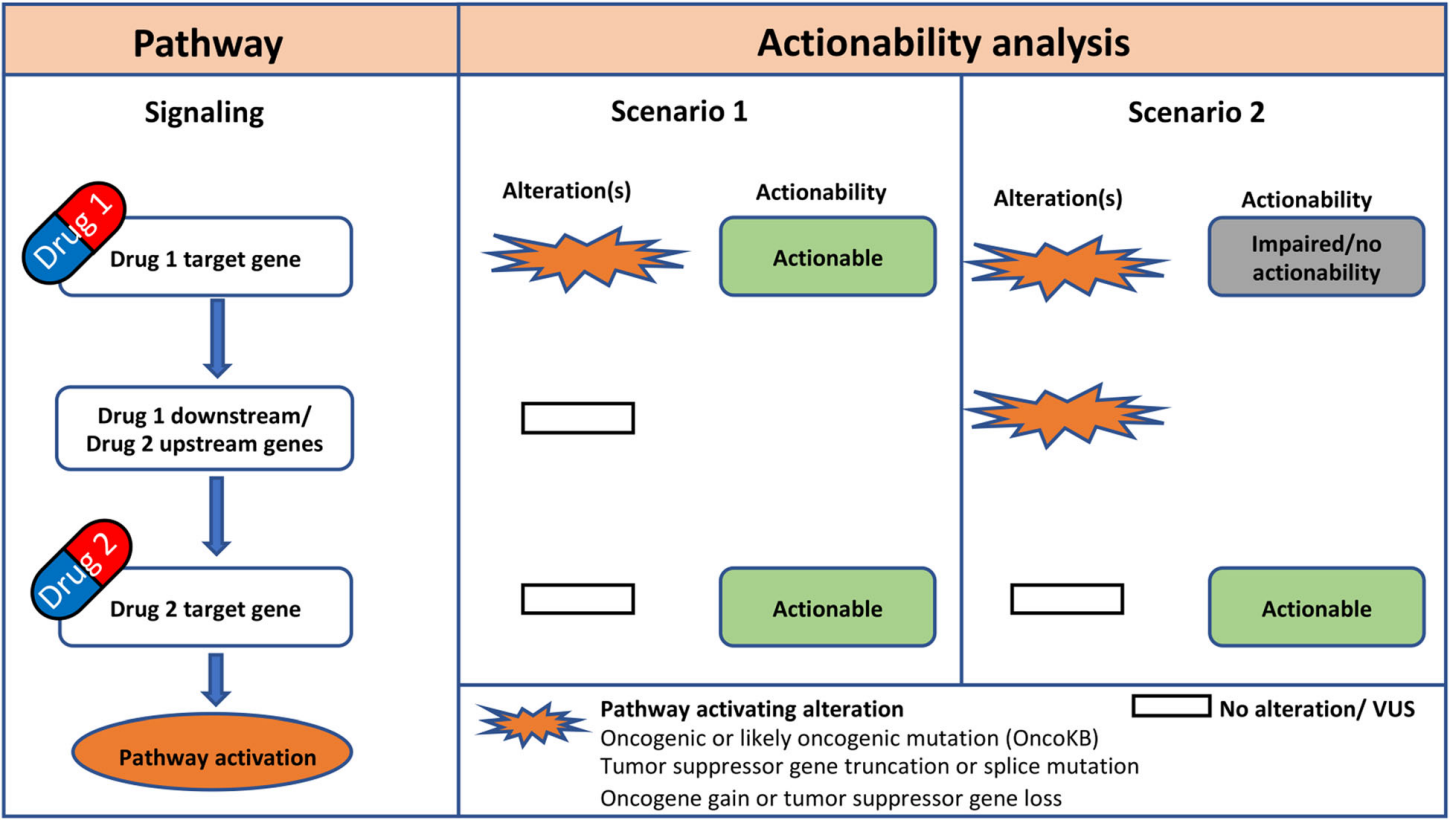
Principles of actionability analysis performed in this study. An illustration of gene actionability analysis is shown for a cancer signaling pathway with two drug target genes

We next sought to determine the proportion of patients with alterations profiles that could indicate actionability of certain cancer drug target genes. Gene actionability was evaluated by a pathway-based approach as illustrated in Fig. 2. For gene actionability to be postulated, alterations had to be detected on or upstream of the drug target gene in a specified signaling pathway, and no alterations could be detected downstream of the drug target gene.

**Fig. 3:**
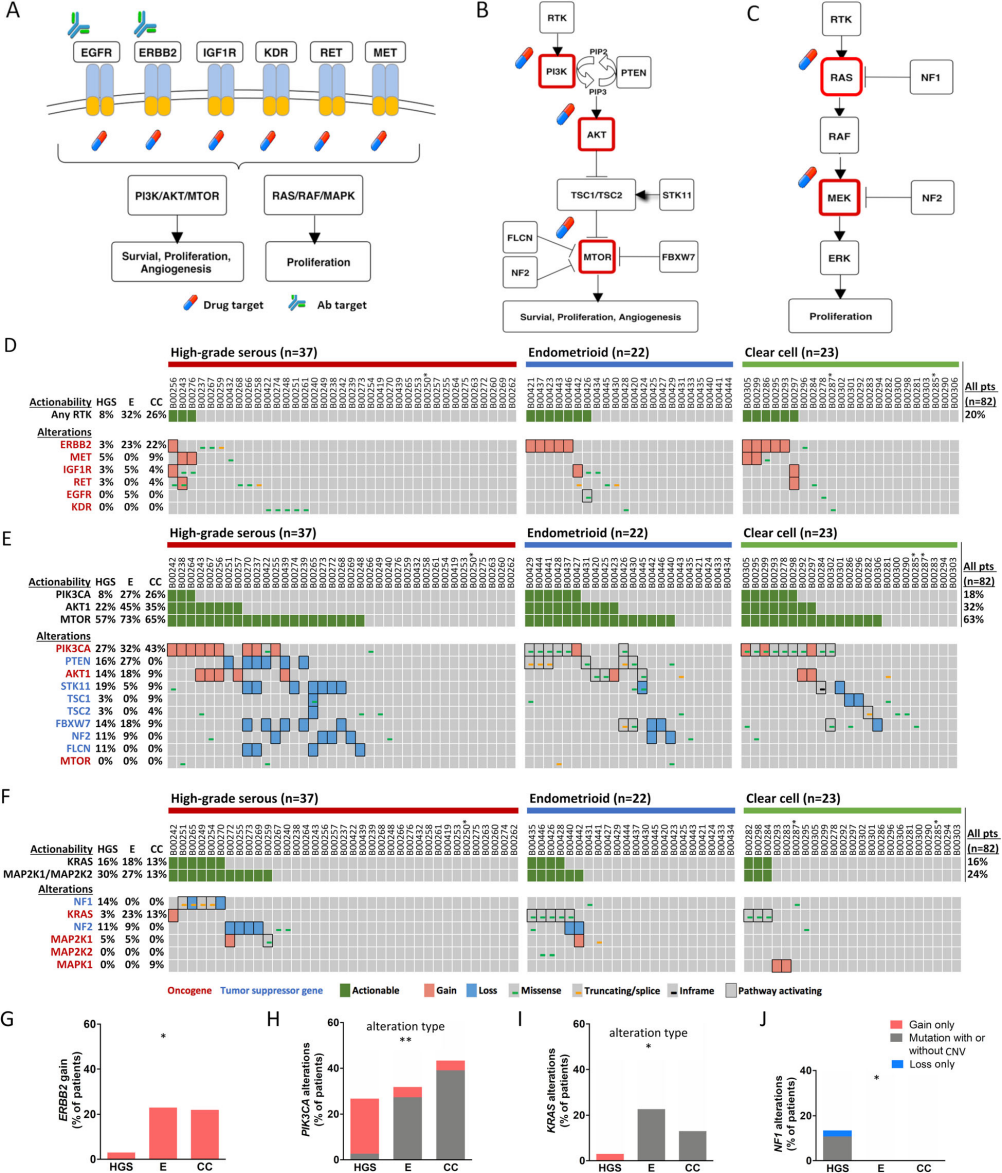
Actionability of genes in receptor tyrosine kinase (RTK)-related pathways. Receptor tyrosine kinases (RTK) (**a**), the PI3K/AKT/MTOR pathway (**b**), and the RAS/MAPK pathway (**c**) and are displayed. Oncoprint plots are depicted for genetic alterations and their postulated actionability in RTK genes (**d**), genes of the PI3K/AKT/MTOR pathway (**e**), and genes of the RAS/MAPK pathway (**f**). The differential distribution of genetic alterations for *ERBB2* (**g**), *PIK3CA* (**h**), *KRAS* (**i**) and *NF1* (**j**) between histological subtypes is shown in bar charts. All statistical analyses were performed by the Chi-Square test (see Additional file 7). Statistical significance is displayed as *P < 0.05, and **P < 0.01. Histological subtypes are abbreviated as HGS = high-grade serous, E = endometrioid and CC = clear cell. *MAP 2 K1* = *MEK1*, *MAP 2 K2* = *MEK2* and *MAPK1* = *ERK2*. In oncoprint plots, * indicates a sample with low (20–30%) tumor purity

We first analyzed the receptor tyrosine kinase (RTK), PI3K/AKT/MTOR and RAS/MAPK pathways as illustrated in Fig. 3a-c. For RTK, the gene with the clearest actionability was *ERBB2*, with gain being observed in 3, 23 and 22% of patients with high-grade serous, endometrioid and clear cell carcinomas, respectively (Fig. 3d). In contrast, only one to four patients in the whole cohort had alterations in *MET, RET, IGF1R* or *EGFR*. Importantly, all alterations were copy number gains, with the exception of an *EGFR* mutation (G588S). We next analyzed the signaling pathways downstream of RTK.

In the PI3K/AKT/MTOR pathway, *PIK3CA* alterations were common in all histological subtypes (Fig. 3e). In contrast, alterations in *PIK3CB, PIK3CG* and *PIK3CD* were rare (Additional file 8). Due to alterations in the other downstream signaling genes, postulated PI3K actionability was lower than would have been anticipated by the sole analysis of *PIK3CA* alterations, and ranged between 8 and 27%, depending on the histological subtype.

AKT1 actionability was 22, 45 and 35% in patients with high-grade serous, endometrioid and clear cell carcinomas, respectively, and *AKT1* alterations were observed in 14–18% of patients with serous and endometrioid carcinomas. *AKT2* and *AKT3* alterations were rare (Additional file 8).

The predicted actionability of MTOR was at least 57% in all histological subtypes.

In the RAS/MAPK pathway, the most dominant oncogene in was *KRAS* (Fig. 3f). There were no alterations of *BRAF*, *NRAS* and *HRAS* (data not shown).

Although not yet established in clinical practice, KRAS is now considered druggable [58]. KRAS actionability was 16, 18 and 13% in patients with high-grade serous, endometrioid and clear cell carcinomas, respectively, whereas MEK1/2 actionability was 30, 27 and 13%.

On a single-gene level, *ERBB2* had only gain as an actionable alteration type. Such *ERBB2* gain was a hallmark of endometrioid and clear cell carcinomas (*P* = 0.036, Fig. 3g). In contrast, *PIK3CA* and *KRAS* were altered by mutations in those histological subtypes, in contrast to high-grade serous carcinomas (*P* = 0.002 and *P* = 0.0495, respectively, Fig. 3h and i, and Additional file 7). The most commonly mutated hotspot for *PIK3CA* was H1047R/L. For *KRAS*, mutations of codons 12 and 13 were observed in seven of the eight patients who harbored *KRAS* mutations (Additional file 6). Regarding *NF1* alterations, 14% (*n* = 5) of high-grade serous carcinoma patients harbored alterations in this gene, whereas no alterations were observed in the other histological subtypes (*P* = 0.039, Fig. 3j).

### Gene actionability of the cell cycle and hedgehog pathways

**Fig. 4:**
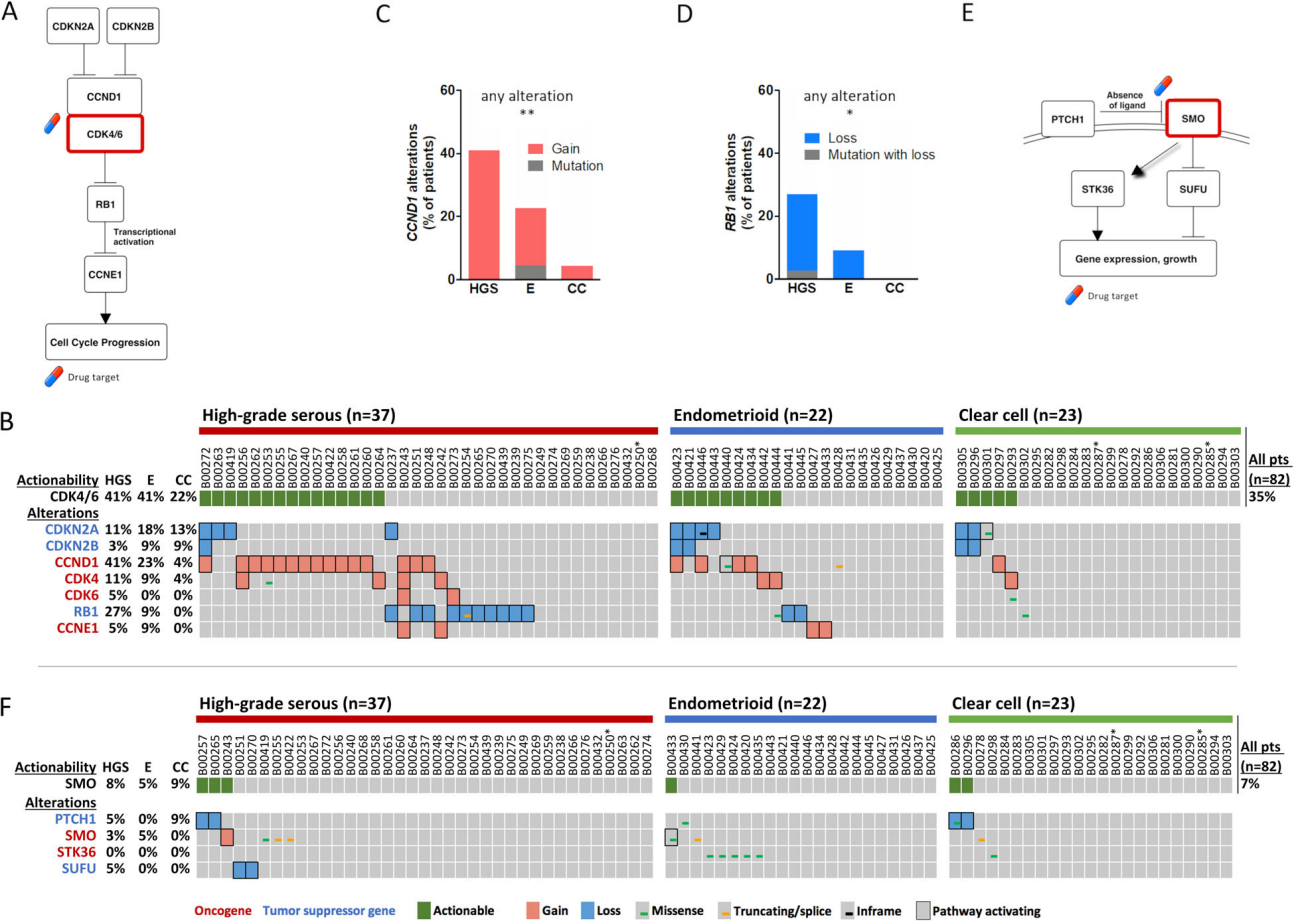
Actionability of cell cycle and hedgehog pathway genes. The cell cycle pathway is displayed (**a**). An oncoprint plot is depicted for genetic alterations in cell cycle pathway genes and postulated CDK4/6 actionability (**b**). The differential distribution of genetic alterations for *CCND1* (**c**) and *RB1* (**d**) between histological subtypes is shown in bar charts. All statistical analyses were performed by the Chi-Square test (Additional file 7). Statistical significance is displayed as *P < 0.05, and **P < 0.01. The hedgehog pathway is shown (**e**). An oncoprint plot is depicted for genetic alterations in hedgehog pathway genes and their postulated actionability (**f**). Histological subtypes are abbreviated as HGS = high-grade serous, E = endometrioid and CC = clear cell. In oncoprint plots, * indicates a sample with low (20–30%) tumor purity

We next analyzed genes involved in the regulation of the cell cycle (Fig. 4a), which was mostly affected by CNV (Fig. 4b).

In high-grade serous carcinomas, *CCND1* gain was the most important alteration for postulated CDK4/6 actionability, and the prevalence of *CCND1* alterations was statistically different between histological subtypes (*P* = 0.007, Fig. 4c and Additional file 7). *CCNE1* gain and *RB1* loss could potentially impair actionability in some patients with high-grade serous carcinomas. However, observed *RB1* inactivating alterations were mostly heterozygous deletions (Additional file 6). Alterations of *RB1* were more prevalent in high-grade serous carcinomas (*P* = 0.011, Fig. 4d and Additional file 7). In total, CDK4/6 actionability in patients with high-grade serous, endometrioid and clear cell carcinomas was 41, 41 and 22%, respectively.

Similar to the cell cycle pathway, the hedgehog pathway with *SMO* as an actionable gene was mostly affected by CNV (Fig. 4e and f). In most patients, this pathway did not harbor postulated actionable alterations.

Lastly, we compared our data of key gene alterations from the different analyzed pathways with the available literature for serous [13, 55, 65, 66], endometrioid [17, 55, 56, 66–68], and clear cell [55, 66, 67, 69] carcinoma patients (Additional file 9). Observed alteration frequencies were mostly comparable.

### Actionability profile in our study cohort

**Fig. 5:**
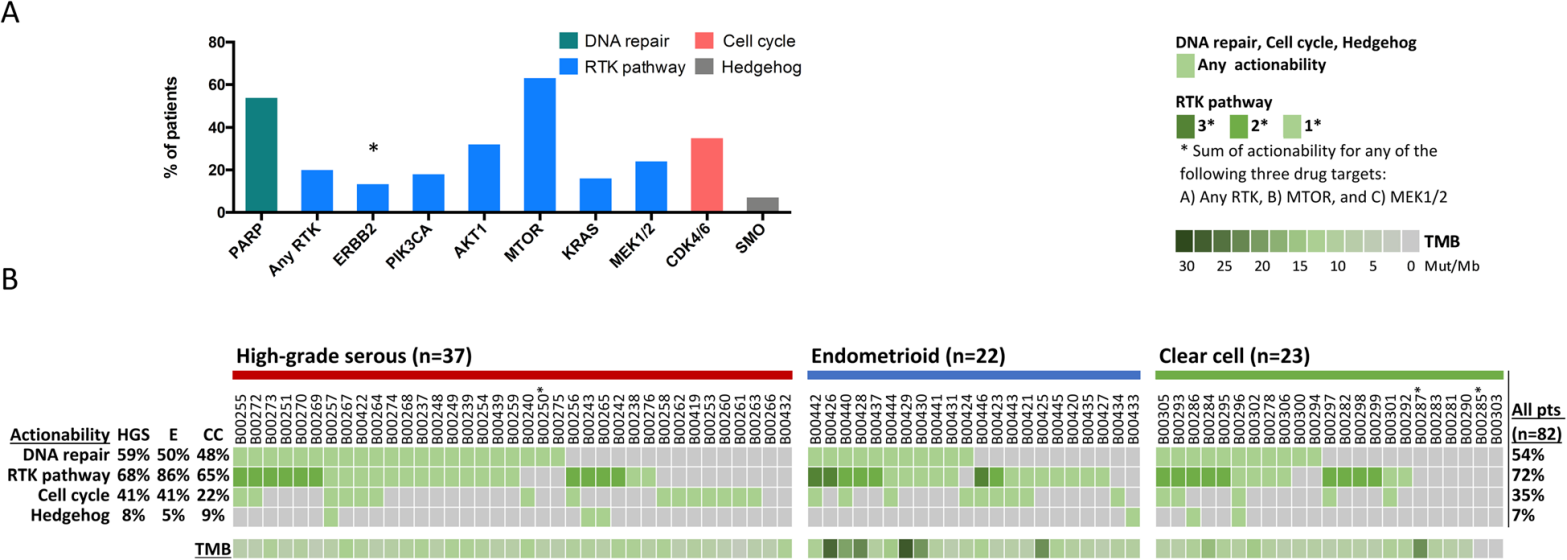
Summary of actionability and tumor mutational burden (TMB) for patients of different histological subtypes. The actionability of alterations in patients is displayed for different cancer signaling pathways/genes for the overall cohort (*n* = 82) (**a**). Only actionabilities for drug targets with at least five patients with potential eligibility for targeted therapy are shown. Statistical analysis was performed by the Chi-Square test for differential distribution of actionability for the displayed drug targets among histological subtypes (see Additional file 10). Statistical significance is displayed as *P < 0.05. An oncoprint plot depicts information for actionability and its overlap, as well as TMB, for individual patients with different histological subtypes (**b**). Histological subtypes are abbreviated as HGS = high-grade serous, E = endometrioid and CC = clear cell. In the oncoprint plot, * indicates a sample with low (20–30%) tumor purity

Finally, we summarized our results. The highest postulated actionability was observed for PARP, MTOR, and CDK4/6. Although, as previously mentioned, the proportions of patients with any alteration of the key genes *ARID1A, BRCA1/2, CCND1, ERBB2, PTEN,* and *RB1* differed between histological subtypes (Additional file 7), ERBB2 was the only drug target with a significantly different actionability distribution (Fig. 5a and Additional file 10).

Almost all patients had at least one postulated actionability (Fig. 5b). There was a high overlap of actionabilities, as patients harbored concurrent alterations in multiple pathways, e.g., in the RTK-related and DNA repair pathways.

For the RTK-related pathways, there was substantial overlap of actionability for RTK and the drug targets downstream of the MTOR pathway (MTOR) and the RAS/MAPK pathway (MEK1/2) (Fig. 5b). Such an overlap was observed in 37–60% of patients with alterations in RTK-related pathways (high-grade serous: 40% (10/25), endometrioid: 37% (7/19), and clear cell: 60% (9/15)).

## Discussion

In the present study, we comprehensively analyzed genetic alterations, including mutations and CNVs, of ovarian tumors with different histologies. We determined the eligibility of patients for targeted therapies. To obtain more accurate results for the determination of treatment sensitivity, we designed a pathway-based analysis to include the evaluation of genetic alteration patterns for individual patients.

As expected, and in accordance with our previous results from a study cohort overlapping with the present cohort, *BRCA1/2* alterations were a hallmark of high-grade serous carcinomas [13, 24]. However, there is growing evidence that alterations of other genes involved in DNA repair and homologous recombination, such as *ARID1A, ATM, CHEK2*, and *PTEN*, occur in a substantial proportion of patients, and can positively influence sensitivity to PARPi [13, 70–74]. For endometrioid and clear cell carcinomas, almost all our patients with postulated PARPi sensitivity harbored *ARID1A* mutations. In line with our results, *ARID1A* mutations have previously been suggested as an interesting actionable alteration for PARPi therapy, especially in clear cell carcinomas [75]. *ARID1A* mutations were mostly private, truncating mutations, and such inactivating mutations presumably result in a loss of function through lack of protein expression [76]. In endometrioid carcinomas, *ARID1A* mutations often co-occurred with *PTEN* mutations, and concurrent loss of those tumor suppressors has been shown to have synergistic effects on tumorigenesis [77]. *PTEN* displayed a very distinct genetic alteration profile among subtypes, being only altered by copy number loss in high-grade serous carcinomas, and by mutations in endometrioid carcinomas. The rarity of *PTEN* mutations in clear cell and serous carcinomas is in accordance with the literature [13, 17, 18]. Notably, *PTEN* copy number loss was mostly due to heterozygous deletion. Such inactivation of one copy of haploinsufficient tumor suppressor genes, e.g., *PTEN* and other genes included in this study [78–82], can have tumorigenic effects. While monoallelic inactivation of haploinsufficient tumor suppressor genes may not have exactly the same effect on pathway activity as biallelic inactivation, a recent study demonstrated that PTEN level reduction of as little as 20% is tumorigenic [83].

High tumor mutational burden (TMB) is associated with enhanced benefit from immunotherapy [84], although there is no consensus about a cutoff value for enhanced benefit from immunotherapy in ovarian cancer patients. We noted a high TMB in some study patients, mostly for endometrioid carcinomas. These results are consistent with the literature [85]. In our cohort, the patients with the second and third highest TMB harbored mismatch-repair (MMR) gene mutations indicative of biallelic inactivation. This observation suggests that at least some patients may meet the indication criteria for the checkpoint inhibitor blockade. The number of potentially eligible patients may have been higher than identified by our approach. We have not performed microsatellite instability testing or MMR IHC, and may, therefore, have missed MSI-H patients with a high TMB due to MLH1 methylation.

The pathway with the most complex alterations in our study was the PI3K/AKT/MTOR pathway. In all histological subtypes, *PIK3CA* harbored alterations in a substantial proportion of patients. In clear cell or endometrioid carcinoma patients, the most frequently observed alterations were mutations. Such mutations are associated with a better response to inhibitors of the PI3K/AKT/MTOR pathway [86, 87]. In high-grade serous carcinoma patients, *PIK3CA* copy number gain was more prevalent than *PIK3CA* mutation. *PTEN* and *PIK3CA* alterations co-occurred in some of our patients, and *PTEN* inactivation may mediate resistance to PI3K inhibition [88, 89]. However, that effect may be inhibitor specific, and *PTEN* loss did not prevent response in a patient from another study [51]. That study did not identify a clear correlation of PI3K inhibitor response with PI3K and PTEN biomarkers, arguing more complex markers than “single mutational events” may be needed [51]. Indeed, therapeutic implications for PI3K inhibitors might vary considerably due to the different downstream patterns of genetic alterations, and patients with alterations in such downstream genes might be more suitable for MTOR inhibitor therapy [90]. In our study, patients of all histological subtypes harbored alterations downstream of PI3K, which were mostly due to CNV in high-grade serous carcinomas. Many alterations that could confer resistance to PI3K inhibition and sensitivity to MTOR inhibition would not have been discovered in an analysis focusing only on mutations in single genes. Our study, therefore, indicates the importance of a pathway-based analysis of all genetic alterations, including mutations and CNVs.

According to our results, PI3K and AKT inhibition may be most promising in clear cell and endometrioid carcinomas, although the different distribution between subtypes was not statistically significant. In line with this notion, AKT inhibition yielded most favorable results in two patients with endometrioid and clear cell histology harboring *PTEN* and *PIK3CA* alterations, respectively [11]. In our study, MTOR actionability was very common in all histological subtypes. Although responses to MTOR inhibition are observed in ovarian cancer patients [53, 54], response rates are relatively low. This may be explained by MTOR independent signaling through PI3K and AKT, and activation of an AKT feedback loop [45]. Therefore, a more specific inhibition may be desirable in PI3K or AKT actionable patients without further downstream alterations.

Another possible resistance mechanism to PI3K/AKT/MTOR inhibition is *RAS* mutation [11]. Combining PI3K inhibitors with MEK (MAP 2 K1/2) inhibitors may be feasible and particularly promising in patients with *RAS* mutations [10]. In our study, we were able to identify *KRAS* mutations in patients with endometrioid and clear cell carcinomas. *KRAS* mutant ovarian cancer patients show responses to MEK inhibitors, however the predictive value of *RAS* mutations differed between studies [12, 91]. A closer biomarker evaluation may shed more light on how to identify responsive patients.

It should be noted that KRAS, while having proven “undruggable” for decades, is now being targeted by inhibitors in clinical studies. Those inhibitors can be mutation-specific or act more broadly, e.g. through inhibiting the KRAS-SOS1 interaction [58]. In that context, it is interesting to note that the *KRAS* alteration profile in our study differed between histological subtypes, with high-grade serous carcinomas harboring only gain in one patient, while endometrioid and clear cell carcinomas harbored only mutations, most of which were located in codons 12 and 13.We observed that in some patients, postulated sensitivity to KRAS or MEK inhibitors was not based on *KRAS* alterations, but on *NF1, NF2* or *MEK* alterations. In particular, *NF1* alterations, which occurred only in high-grade serous carcinomas in our cohort, are underappreciated as actionable alterations in this histology. However, a few recent ovarian cancer case reports indicate their potential as biomarkers for MEK inhibitors [92, 93], and encouraging preclinical data are available for KRAS inhibitors [94]. Remarkably, *NF1* was preferentially altered by mutation and not copy number alteration. This further indicates a role as a major driver of malignancy in high-grade serous carcinoma, in which some cancer gene CNV may be byproducts of a copy number unstable genetic profile.

A drug target that has not been included in this study is RAF, and there were no *BRAF* alterations in our study cohort. However, while most inhibitors are specifically inhibiting mutated BRAF and would therefore be limited to RAF-mutant patients, it should be noted that next-generation RAF inhibitors also inhibit wildtype BRAF and are effective in RAS-mutant cells [95].

In conclusion, our results indicate that patients could benefit from broader profiling to determine eligibility for RAS/MAPK pathway inhibitors, and may be candidates for emerging targeted therapies such as KRAS and BRAF inhibitors.

Outcomes for ovarian cancer patients treated with agents targeting receptor tyrosine kinases (RTK) have often been disappointing. ERBB2 was the only RTK that was deemed actionable in a considerable proportion of patients in our cohort, mostly in endometrioid and clear cell carcinomas. It was also the only drug target with a statistically different actionability distribution among histological subtypes. Genetic *ERBB2* alterations may be an interesting biomarker considering the rather disappointing results for ERBB2 targeting antibodies, and the limited applicability of discussed mRNA biomarkers [40, 41]. Potentially actionable alterations in other investigated RTK were rare in our cohort, however, broad genetic testing may identify those RTK as potential actionable targets in a minority of patients.

A factor limiting the effectivity of RTK inhibitors may be the occurrence of other RTK pathway-related alterations. The combination of RTK-related pathway inhibitors appears highly interesting considering that 37–60% of patients of all histological subtypes with RTK-pathway related alterations had an overlapping postulated gene actionability. A substantial overlap between those pathways is in line with the results from other studies [13, 14].

Postulated sensitivity to CDK4/6 inhibitor therapy was mostly due to *CCND1* gain – particularly in patients with high-grade serous carcinomas- and *CDKN2A* loss. *RB1* loss was a characteristic of high-grade serous carcinomas, and potentially limited postulated CDK4/6 actionability in some high-grade serous carcinoma patients. However, while *RB1* loss-of function mutation has been associated with adaptive resistance to CDK4/6 inhibitors in breast cancers [96], less is known about a potential effect of heterozygous RB1 copy number loss. Since heterozygous *RB1* loss was the most common detected *RB1* inactivating alteration, the role of *RB1* in mediating primary resistance may be limited.

In a trial enrolling mostly patients with serous ovarian carcinomas, the response rate to CDK4/6 inhibition was only 4% using RECIST criteria, although stable disease was commonly observed [60]. We observed a high overlap between actionabilities of RTK-related and cell cycle pathways. Therefore, in some patients, combination therapies may be an interesting approach, which is currently evaluated in the clinic [97].

Another potential candidate for targeted therapy is SMO. However, in our cohort, we postulated actionability in only 5–9% of patients, depending on the histological subtype. This is consistent with disappointing results of the SMO inhibitor vismodegib in ovarian cancer patients [8].

Genetic alteration frequencies detected in key genes in this cohort and those reported in the literature were mostly comparable (Additional file 9). However, for some gene alterations, literature results varied, were unavailable, or based on small patient groups. Therefore, future studies are eagerly awaited. When further comparing our high-grade serous carcinoma patients and the comprehensive TCGA dataset, we observed some similarities, including the domination of CNV in the genetic alteration landscape and a substantial overlap of alterations between RTK-related pathways in individual patients.

It should be noted that there is a need to further investigate the therapeutic implications of concurrent actionable alterations in multiple pathways. This is particularly relevant in late stage patients with multiple concurrent alterations. Frequent overlapping actionabilities in our cohort between RTK-related, DNA repair and cell cycle pathways indicate a high potential of a combination of targeted therapies. Endometrioid carcinoma patients had a high prevalence of concurrent mutations of *ARID1A, PTEN* and *PIK3CA* and could therefore be easily identifiable candidates for a combination of inhibitors of PARP and the MTOR pathway. Concurrent intra-pathway alterations could also indicate a potential for combination therapies of upstream and downstream drug targets.

An important limitation of our study is the necessity to verify the applicability of our pathway-based analysis by clinical and preclinical data. Such studies could also further analyze the impact of a heterozygous deletion of included haploinsufficient tumor suppressors compared to other alterations. Furthermore, it may be beneficial to supplement genetic alteration analysis by other testing, such as IHC or mRNA expression analysis. Another limitation is the moderate cohort size. However, the study was designed to compare simple categorical outcomes, e.g., whether patients were eligible for targeted treatments, or whether they harbored certain genetic alterations. This reduced the complexity of the analysis to allow for meaningful comparisons despite the limited cohort size. We also acknowledge that a pathway postulated to be activated may not be the main driver of cell growth in some patients. Additionally, a few variants detected in druggable genes, such as *EGFR* (G588S) or *SMO* (I408V), do currently not have an available corresponding drug, or are associated with resistance to available inhibitors [98]. However, for patients with such variants, new inhibitors or alternative agents interfering with new downstream targets may become available in the future [99].

## Conclusions

The present study demonstrates that patients with high-grade serous, endometrioid and clear cell ovarian carcinomas differ in their tumor genetic profiles. Those findings have implications for the personalization of therapies as well as patient selection for clinical studies. Although underlining the potential of comprehensive genetic testing, this study may also provide guidance regarding the selection of candidate genes for genetic testing.

## Supplementary Information

**Additional file 1.** Complete list of all 410 genes sequenced in the present study.

**Additional file 2.** List of mean sequencing depth and uniformity for all study patients for both cancer panels used in the present study.

**Additional file 3.** Design and work flow of the present study. E = endometrioid, CC = clear cell, CNV = copy number variation, FFPE = formalin-fixed paraffin-embedded, HGS = high-grade serous, MMR = mismatch repair NGS = next-generation sequencing, SNV = single nucleotide variant, and TMB = tumor mutational burden.

**Additional file 4.** Patient characteristics of ovarian cancer patients included in the present study.

**Additional file 5.** Protein coding and splice mutations observed in all patients included in the present study (*n* = 82).

**Additional file 6.** Detected mutation and copy number variants in analyzed cancer signaling pathways, and their classification.

**Additional file 7.** Genetic alterations and TMB in ovarian cancer patients of different histological subtypes.

**Additional file 8.** Specific genetic alterations of the PI3K/AKT/MTOR pathway. Oncoprint plots are depicted for the genes *PIK3CA/B/G/D* and *AKT1/2/3* according to histological subtypes. Histological subtypes are abbreviated as HGS = high-grade serous, E = endometrioid and CC = clear cell. * indicates a sample with low (20–30%) tumor purity.

**Additional file 9.** Comparison of key gene alteration frequencies in the present study and in the literature.

**Additional file 10.** Postulated actionability for analyzed pathways in ovarian cancer patients of different histological subtypes.

## Data Availability

The datasets used and/or analyzed during the current study are available from the corresponding author on reasonable request.

## Abbreviations

CNV: Copy number variation
FFPE: Formalin-fixed paraffin-embedded
PARPi: Poly (ADP-ribose) polymerase inhibitor
RTK: Receptor tyrosine kinase
TMB: Tumor mutational burden
VUS: Variant of unknown significance
