# Supplementary material for: Genetic alterations and their therapeutic implications in epithelial ovarian cancer
Source: BMC Cancer. 2021 May 4;21:499. doi: 10.1186/s12885-021-08233-5 (PMC8097933; doi:10.1186/s12885-021-08233-5)
Supplement: Supplementary file 1 — Additional file 1. Complete list of all 410 genes sequenced in the present study. [file 12885_2021_8233_MOESM1_ESM.docx]

**Additional file 1.** Complete list of all 410 genes sequenced in the present study.

| ABL1 | BUB1B | DST | G6PD | KLF6 | MYCN | PKHD1 | SMAD4 | WAS |
| --- | --- | --- | --- | --- | --- | --- | --- | --- |
| ABL2 | CARD11 | EGFR | GATA1 | KMT2B | MYD88 | PLAG1 | SMARCA4 | WHSC1 |
| ACVR2A | CASC5 | EML4 | GATA2 | KMT2C | MYH11 | PLCG1 | SMARCB1 | WRN |
| ADAMTS20 | CBL | EP300 | GATA3 | KMT2D | MYH9 | PLEKHG5 | SMO | WT1 |
| AFF1 | CCND1 | EP400 | GDNF | KRAS | NBN | PML | SMUG1 | XPA |
| AFF3 | CCND2 | EPHA3 | GNA11 | LAMP1 | NCOA1 | PMS1 | SOCS1 | XPC |
| AKAP9 | CCNE1 | EPHA7 | GNAQ | LCK | NCOA2 | PMS2 | SOX11 | XPO1 |
| AKT1 | CD79A | EPHB1 | GNAS | LIFR | NCOA4 | POT1 | SOX2 | XRCC2 |
| AKT2 | CD79B | EPHB4 | GPR124 | LPHN3 | NF1 | POU5F1 | SRC | ZNF384 |
| AKT3 | CDC73 | EPHB6 | GRM8 | LPP | NF2 | PPARG | SSX1 | ZNF521 |
| ALK | CDH1 | ERBB2 | GUCY1A2 | LRP1B | NFE2L2 | PPP2R1A | STK11 |  |
| AMER1 | CDH11 | ERBB3 | HCAR1 | LTF | NFKB1 | PRDM1 | STK36 |  |
| APC | CDH2 | ERBB4 | HIF1A | LTK | NFKB2 | PRKAR1A | SUFU |  |
| AR | CDH20 | ERCC1 | HLF | MAF | NIN | PRKDC | SYK |  |
| ARID1A | CDH5 | ERCC2 | HNF1A | MAFB | NKX2-1 | PSIP1 | SYNE1 |  |
| ARID2 | CDK12 | ERCC3 | HOOK3 | MAGEA1 | NLRP1 | PTCH1 | TAF1 |  |
| ARNT | CDK4 | ERCC4 | HRAS | MAGI1 | NOTCH1 | PTEN | TAF1L |  |
| ASXL1 | CDK6 | ERCC5 | HSP90AA1 | MALT1 | NOTCH2 | PTGS2 | TAL1 |  |
| ATF1 | CDK8 | ERG | HSP90AB1 | MAML2 | NOTCH4 | PTPN11 | TBX22 |  |
| ATM | CDKN2A | ESR1 | ICK | MAP2K1 | NPM1 | PTPRD | TCF12 |  |
| ATR | CDKN2B | ETS1 | IDH1 | MAP2K2 | NRAS | PTPRT | TCF3 |  |
| ATRX | CDKN2C | ETV1 | IDH2 | MAP2K4 | NSD1 | RAD50 | TCF7L1 |  |
| AURKA | CEBPA | ETV4 | IGF1R | MAP3K7 | NTRK1 | RAF1 | TCF7L2 |  |
| AURKB | CHEK1 | EXT1 | IGF2 | MAPK1 | NTRK3 | RALGDS | TCL1A |  |
| AURKC | CHEK2 | EXT2 | IGF2R | MAPK8 | NUMA1 | RARA | TET1 |  |
| AXL | CIC | EZH2 | IKBKB | MARK1 | NUP214 | RB1 | TET2 |  |
| BAI3 | CKS1B | FANCA | IKBKE | MARK4 | NUP98 | RECQL4 | TFE3 |  |
| BAP1 | CMPK1 | FANCC | IKZF1 | MBD1 | PAK3 | REL | TGFBR2 |  |
| BCL10 | COL1A1 | FANCD2 | IL2 | MCL1 | PALB2 | RET | TGM7 |  |
| BCL11A | CRBN | FANCF | IL21R | MDM2 | PARP1 | RHOH | THBS1 |  |
| BCL11B | CREB1 | FANCG | IL6ST | MDM4 | PAX3 | RNASEL | TIMP3 |  |
| BCL2 | CREBBP | FAS | IL7R | MEN1 | PAX5 | RNF2 | TLR4 |  |
| BCL2L1 | CRKL | FBXW7 | ING4 | MET | PAX7 | RNF213 | TLX1 |  |
| BCL2L2 | CRTC1 | FGFR1 | IRF4 | MITF | PAX8 | ROS1 | TNFAIP3 |  |
| BCL3 | CSF1R | FGFR2 | IRS2 | MLH1 | PBRM1 | RPS6KA2 | TNFRSF14 |  |
| BCL6 | CSMD3 | FGFR3 | ITGA10 | MLLT10 | PBX1 | RRM1 | TNK2 |  |
| BCL9 | CTNNA1 | FGFR4 | ITGA9 | MMP2 | PDGFB | RUNX1 | TOP1 |  |
| BCR | CTNNB1 | FH | ITGB2 | MN1 | PDGFRA | RUNX1T1 | TP53 |  |
| BIRC2 | CYLD | FLCN | ITGB3 | MPL | PDGFRB | SAMD9 | TPR |  |
| BIRC3 | CYP2C19 | FLI1 | JAK1 | MRE11A | PER1 | SBDS | TRIM24 |  |
| BIRC5 | CYP2D6 | FLT1 | JAK2 | MSH2 | PGAP3 | SDHA | TRIM33 |  |
| BLM | DAXX | FLT3 | JAK3 | MSH6 | PHOX2B | SDHB | TRIP11 |  |
| BLNK | DCC | FLT4 | JUN | MTOR | PIK3C2B | SDHC | TRRAP |  |
| BMPR1A | DDB2 | FN1 | KAT6A | MTR | PIK3CA | SDHD | TSC1 |  |
| BRAF | DDIT3 | FOXL2 | KAT6B | MTRR | PIK3CB | SEPT9 | TSC2 |  |
| BRCA1^a^ | DDR2 | FOXO1 | KDM5C | MUC1 | PIK3CD | SETD2 | TSHR |  |
| BRCA2^a^ | DEK | FOXO3 | KDM6A | MUTYH | PIK3CG | SF3B1 | UBR5 |  |
| BRD3 | DICER1 | FOXP1 | KDR | MYB | PIK3R1 | SGK1 | UGT1A1 |  |
| BRIP1 | DNMT3A | FOXP4 | KEAP1 | MYC | PIK3R2 | SH2D1A | USP9X |  |
| BTK | DPYD | FZR1 | KIT | MYCL | PIM1 | SMAD2 | VHL |  |

^a^ The genes *BRCA1/2* were analyzed in a separate test.
