# Supplementary material for: Genetic alterations and their therapeutic implications in epithelial ovarian cancer
Source: BMC Cancer. 2021 May 4;21:499. doi: 10.1186/s12885-021-08233-5 (PMC8097933; doi:10.1186/s12885-021-08233-5)
Supplement: Supplementary file 3 — Additional file 3. Design and work flow of the present study. E = endometrioid, CC = clear cell, CNV = copy number variation, FFPE = formalin-fixed paraffin-embedded, HGS = high-grade serous, MMR = mismatch repair NGS = next-generation sequencing, SNV = single nucleotide variant, and TMB = tumor mutational burden. [file 12885_2021_8233_MOESM3_ESM.pdf]

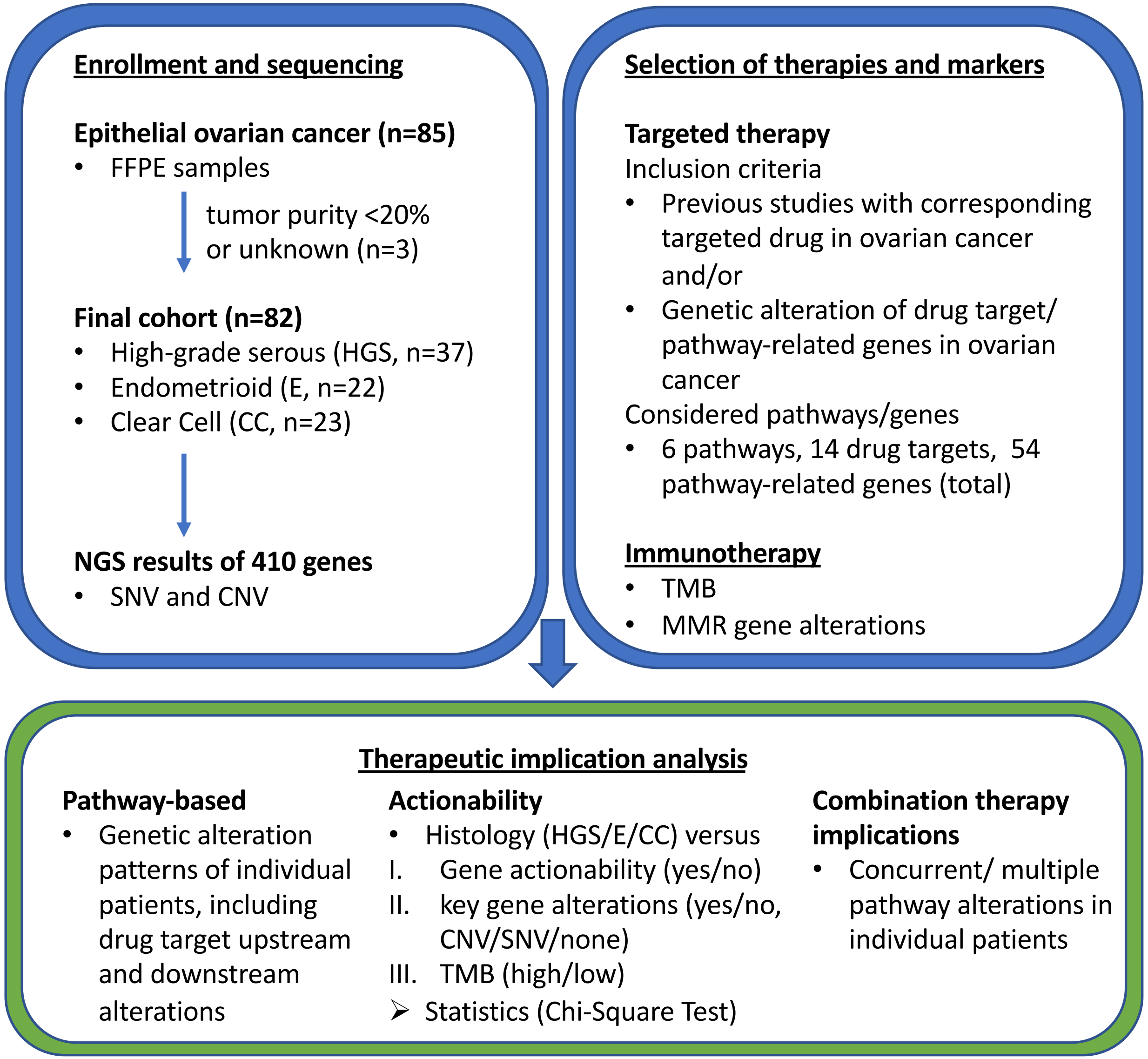
Enrollment and sequencing
Epithelial ovarian cancer (n=85)
• FFPE samples
tumor purity <20% or unknown (n=3)
Final cohort (n=82)
• High-grade serous (HGS, n=37)
• Endometrioid (E, n=22)
• Clear Cell (CC, n=23)
NGS results of 410 genes
• SNV and CNV
Selection of therapies and markers
Targeted therapy
Inclusion criteria
• Previous studies with corresponding targeted drug in ovarian cancer and/or
• Genetic alteration of drug target/ pathway-related genes in ovarian cancer
Considered pathways/genes
• 6 pathways, 14 drug targets, 54 pathway-related genes (total)
Immunotherapy
• TMB
• MMR gene alterations
Therapeutic implication analysis
Pathway-based
• Genetic alteration patterns of individual patients, including drug target upstream and downstream alterations
Actionability
• Histology (HGS/E/CC) versus
I. Gene actionability (yes/no)
II. key gene alterations (yes/no, CNV/SNV/none)
III. TMB (high/low)
➢ Statistics (Chi-Square Test)
Combination therapy implications
• Concurrent/ multiple pathway alterations in individual patients
