# Supplementary material for: Genetic alterations and their therapeutic implications in epithelial ovarian cancer
Source: BMC Cancer. 2021 May 4;21:499. doi: 10.1186/s12885-021-08233-5 (PMC8097933; doi:10.1186/s12885-021-08233-5)
Supplement: Supplementary file 4 — Additional file 4. Patient characteristics of ovarian cancer patients included in the present study. [file 12885_2021_8233_MOESM4_ESM.docx]

**Additional file 4.** Patient characteristics of ovarian cancer patients included in the present study.

|  |  | Entire cohort | High-grade serous | Endometrioid | Clear cell |
| --- | --- | --- | --- | --- | --- |
|  |  | **n (%)** | **n (%)** | **n (%)** | **n (%)** |
|  |  | 82 (100) | 37 (45) | 22 (27) | 23 (28) |
| Age, years |  |  |  |  |  |
|  | Median | 52 | 53 | 47 | 53 |
|  | Range | 23-83 | 40-83 | 23-73 | 26-66 |
|  | Mean ± SD | 53.1 ± 11.1 | 57.0 ± 11.5 | 48.0 ± 10.5 | 51.9 ± 8.8 |
| FIGO stage |  |  |  |  |  |
|  | I, II | 29 (35)^b^ | 3 (10)^d^ | 12 (41)^d^ | 14 (48)^d^ |
|  | III, IV | 53 (65)^b^ | 34 (64)^e^ | 10 (19)^e^ | 9 (17)^e^ |
| Grade^a^ |  |  |  |  |  |
|  | 1 | 3 (5) ^c^ | 0 (0)^f^ | 3 (100)^f^ | NA |
|  | 2,3 | 56 (95) ^c^ | 37 (66)^g^ | 19 (34)^g^ | NA |
| Recurrence |  |  |  |  |  |
|  | No | 26 (32)^b^ | 5 (19)^h^ | 12 (46)^h^ | 9 (35)^h^ |
|  | Yes | 55 (67)^b^ | 32 (58)^i^ | 10 (18)^i^ | 13 (24)^i^ |
|  | Unknown | 1 (1)^b^ | 0 (0)^j^ | 0 (0)^j^ | 1 (100)^j^ |

^a^ No grading was performed for clear cell carcinoma.

^b^ The percentage was calculated in relation to the entire cohort (n=82).

^c^ The percentage was calculated in relation to all patients for whose tumors grading was performed (n=59).

^d^ The percentage was calculated in relation to all patients with FIGO stages I and II (n=29)

^e^ The percentage was calculated in relation to all patients with FIGO stages III and IV (n=53).

^f^ The percentage was calculated in relation to all patients with grade 1 tumors (n =3).

^g^ The percentage was calculated in relation to all patients with grade 2–3 tumors (n=56).

^h^ The percentage was calculated in relation to all patients without disease recurrence (n=26).

^i^ The percentage was calculated in relation to all patients with disease recurrence (n=55).

^j^ The percentage was calculated in relation to all patients without known disease recurrence status (n=1).

NA, not applicable
