# Supplementary material for: Genetic alterations and their therapeutic implications in epithelial ovarian cancer
Source: BMC Cancer. 2021 May 4;21:499. doi: 10.1186/s12885-021-08233-5 (PMC8097933; doi:10.1186/s12885-021-08233-5)
Supplement: Supplementary file 7 — Additional file 7. Genetic alterations and TMB in ovarian cancer patients of different histological subtypes. [file 12885_2021_8233_MOESM7_ESM.docx]

**Additional file 7.** Genetic alterations and TMB in ovarian cancer patients of different histological subtypes.

|  | | All patients | High-grade serous | Endometrioid | Clear cell | P value^a^ |
| --- | --- | --- | --- | --- | --- | --- |
|  |  | **n (%)** | **n (%)^b^** | **n (%)^b^** | **n (%)^b^** |  |
|  |  | **82 (100)** | **37 (45)^c^** | **22 (27)^c^** | **23 (28)^c^** |  |
| Detection of any alteration^d^ | | | | | | |
| *ARID1A* | Yes | 17 (21) | 1 (3) | 7 (32) | 9 (39) | 0.001 |
|  | No | 65 (79) | 36 (97) | 15 (68) | 14 (61) |  |
| *BRCA1/2* | Yes | 11 (13) | 11 (30) | 0 (0) | 0 (0) | <0.001 |
|  | No | 71 (87) | 26 (70) | 22 (100) | 23 (100) |  |
| *CCND1* | Yes | 21 (26) | 15 (41) | 5 (23) | 1 (4) | 0.007 |
|  | No | 61 (74) | 22 (59) | 17 (77) | 22 (96) |  |
| *ERBB2* | Yes | 11 (13) | 1 (3) | 5 (23) | 5 (22) | 0.036 |
|  | No | 71 (87) | 36 (97) | 17 (77) | 18 (78) |  |
| *KRAS* | Yes | 9 (11) | 1 (3) | 5 (23) | 3 (13) | 0.055 |
|  | No | 73 (89) | 36 (97) | 17 (77) | 20 (87) |  |
| *PIK3CA* | Yes | 27 (33) | 10 (27) | 7 (32) | 10 (43) | 0.416 |
|  | No | 55 (67) | 27 (73) | 15 (68) | 13 (57) |  |
| *PTEN* | Yes | 12 (15) | 6 (16) | 6 (27) | 0 (0) | 0.033 |
|  | No | 70 (85) | 31 (84) | 16 (73) | 23 (100) |  |
| *RB1* | Yes | 12 (15) | 10 (27) | 2 (9) | 0 (0) | 0.011 |
|  | No | 70 (85) | 27 (73) | 20 (91) | 23 (100) |  |
| Genetic alteration type^d,e^ | | | | | | |
| *KRAS* | Mutation | 8 (10) | 0 (0) | 5 (23) | 3 (13) | 0.0495 |
|  | Gain | 1 (1) | 1 (3) | 0 (0) | 0 (0) |  |
|  | No alteration | 73 (89) | 36 (97) | 17 (77) | 20 (87) |  |
| *PIK3CA* | Mutation w/o gain | 16 (20) | 1 (3) | 6 (27) | 9 (39) | 0.002 |
|  | Gain only | 11 (13) | 9 (24) | 1 (5) | 1 (4) |  |
|  | No alteration | 55 (67) | 27 (73) | 15 (68) | 13 (57) |  |
| *PTEN* | Mutation | 6 (7) | 0 (0) | 6 (27) | 0 (0) | <0.001 |
|  | Loss | 6 (7) | 6 (16) | 0 (0) | 0 (0) |  |
|  | No alteration | 70 (85) | 31 (84) | 16 (73) | 23 (100) |  |
| TMB | | | | | | |
| Median (range), Mut/Mb | | 9.6  (3.2-28.1) | 8.8  (3.2-13.6) | 9.6  (6.4-28.1) | 8.8  (7.2-21.6) |  |
| >18 Mut/Mb | Yes | 7 (9) | 0 (0) | 6 (27) | 1 (4) | 0.001 |
|  | No | 75 (91) | 37 (100) | 16 (73) | 22 (96) |  |

^a^ The P value was calculated by the Chi-Square test.

**^b^** The percentage was calculated in relation to all patients of the respective histological subtype (high-grade serous n=37, endometrioid n=22, clear cell n=23), if not otherwise indicated.

^c^ The percentage was calculated in relation to the overall cohort (n=82).

^d^ The analysis was performed for genes if alterations, not including VUS, occurred in at least 20% of patients of any histological subtype. VUS were not considered for the analysis.

^e^ The analysis was performed only if 1) mutations with or without CNV and CNV only were each observed in at least three patients in the overall cohort, or 2) all patients of a certain histological subtype harbored the same alteration type.

TMB, tumor mutational burden
