# Supplementary figures and images for: Genetic alterations and their therapeutic implications in epithelial ovarian cancer
Source: BMC Cancer. 2021 May 4;21:499. doi: 10.1186/s12885-021-08233-5 (PMC8097933; doi:10.1186/s12885-021-08233-5)

# High-grade serous (n=37)

# Endometrioid (n=22)

# Clear cell (n=23)

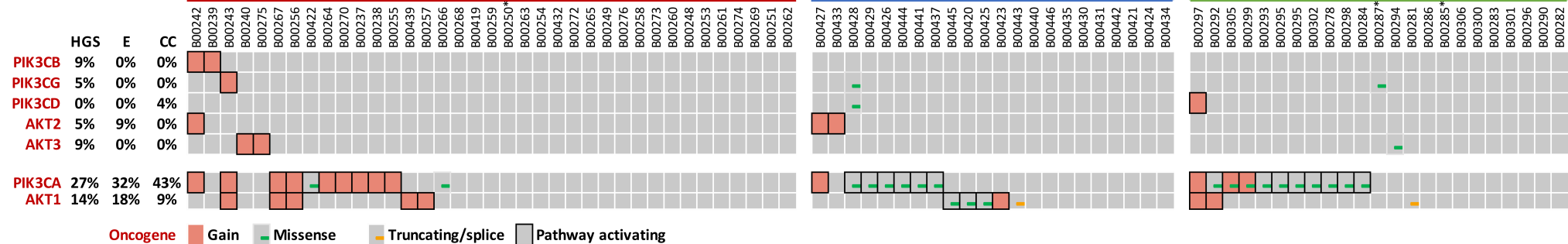

Supplement: Supplementary file 8 — Additional file 8. Specific genetic alterations of the PI3K/AKT/MTOR pathway. Oncoprint plots are depicted for the genes PIK3CA/B/G/D and AKT1/2/3 according to histological subtypes. Histological subtypes are abbreviated as HGS = high-grade serous, E = endometrioid and CC = clear cell. * indicates a sample with low (20–30%) tumor purity. [file 12885_2021_8233_MOESM8_ESM.pdf]
