# Supplementary material for: Genetic alterations and their therapeutic implications in epithelial ovarian cancer
Source: BMC Cancer. 2021 May 4;21:499. doi: 10.1186/s12885-021-08233-5 (PMC8097933; doi:10.1186/s12885-021-08233-5)
Supplement: Supplementary file 9 — Additional file 9. Comparison of key gene alteration frequencies in the present study and in the literature. [file 12885_2021_8233_MOESM9_ESM.docx]

**Additional file 9.** Comparison of key gene alteration frequencies in the present study and in the literature.

|  | | Serous^a^ | | Endometrioid | | Clear cell | |
| --- | --- | --- | --- | --- | --- | --- | --- |
|  | | Mutation | CNV^b^ | Mutation | CNV^b^ | Mutation | CNV^b^ |
| *ARID1A* | Literature | 1% | 1% | 20-30% | NA | 46-62% | NA |
|  | Present study | 3% | 0% / 0% | 32% | 0% / 0% | 39% | 0% / 0% |
| *BRCA1/2* | Literature | 22% | 1% | 6-10% | NA | 6% | NA |
|  | Present study | 27% | 0% / 3% | 0% | 0% / 0% | 0% | 0% / 0% |
| *CCND1* | Literature | 0% | 4-72%^c^ | NA | 33%^d^ | NA | 0%^d^ |
|  | Present study | 0% | 41% | 5% | 18% | 0% | 4% |
| *ERBB2* | Literature | 0-1% | 0-1% | 0% | 10%^e^ | 0% | 0-33% |
|  | Present study | 0% | 3% | 0% | 23% | 0% | 22% |
| *KRAS* | Literature | 0-1% | 0-11% | 15-29% | 0%^e^ | 0-26% | 0-18% |
|  | Present study | 0% | 3% | 23% | 0% | 13% | 0% |
| *PIK3CA* | Literature | 0-1% | 0-17% | 12-32% | 0%^e^ | 29-51% | 0-26% |
|  | Present study | 3% | 24% | 26% | 4% | 39% | 14% |
| *PTEN* | Literature | 1-5% | 0-7% | 31-45% | 0%^e^ | 0-5% | 0% |
|  | Present study | 0% | 3% / 16% | 27% | 0% / 0% | 0% | 0% / 0% |
| *RB1* | Literature | 0-2% | 0-8% | 0%^e^ | 0%^e^ | 0% | 4% |
|  | Present study | 3% | 3% / 27% | 0% | 0% / 9% | 0% | 0% / 0% |

Included literature: serous [13, 55, 65, 66], endometrioid [17, 55, 56, 66-68], and clear cell [55, 66, 67, 69]

^a^ The TCGA cohort [13] similar to our cohort, included only high-grade samples. The cohort of Diebold et al. [65] included a vast majority of serous samples, mostly of grade 2/3. For the cohort of Dimova et al. [66], the proportion of high-grade samples among serous patients is unknown, however, *CCND1* gain was shown to not be associated with grade. For the cohort of Takenaka et al. [55], only patients with Grade 2/3 serous carcinoma were considered in this analysis (n=20).

^b^ For the genes *ARID1A, BRCA1/2, PTEN* and *RB1*, literature data refer to homozygous/deep deletions. Copy number (CN) results from the present study are listed as CN=0.5 (possible homozygous deletion) / CN≤1 (any copy number deletion). CN=0.5 was only observed in two of the indicated genes, namely, *PTEN* (B00439, serous), and *RB1* (B00237, serous). There were no genes with CN=0 in our cohort for the listed genes.

^c^ The comparison shows widely different rates of copy number gain for *CCND1*, ranging between 4% in the TCGA cohort [13] and 72% in the cohort of Diebold et al. [65] However, in the study by Diebold et al. [65], the authors note that most copy number gains in their cohort were small.

^d^ Small sample size; endometrioid, n=15; clear cell, n=10 (Dimova et al. [66]).

^e^ Small sample size; endometrioid, n=10 (Takenaka et al. [55]).

CNV, copy number variant

NA, not applicable.
