# Supplementary material for: Genetic alterations and their therapeutic implications in epithelial ovarian cancer
Source: BMC Cancer. 2021 May 4;21:499. doi: 10.1186/s12885-021-08233-5 (PMC8097933; doi:10.1186/s12885-021-08233-5)
Supplement: Supplementary file 10 — Additional file 10. Postulated actionability for analyzed pathways in ovarian cancer patients of different histological subtypes. [file 12885_2021_8233_MOESM10_ESM.docx]

**Additional file 10.** Postulated actionability for analyzed pathways in ovarian cancer patients of different histological subtypes.

|  | | All patients | High-grade serous | Endometrioid | Clear cell | P value^a^ |
| --- | --- | --- | --- | --- | --- | --- |
|  |  | n (%) | n (%)^b^ | n (%)^b^ | n (%)^b^ |  |
|  |  | 82 (100) | 37 (45) ^c^ | 22 (27) ^c^ | 23 (28) ^c^ |  |
| DNA repair | | | | | | |
| PARP | Yes | 44 (54) | 22 (59) | 11 (50) | 11 (48) | 0.627 |
|  | No | 38 (46) | 15 (41) | 11 (50) | 12 (52) |  |
| RTK pathway^d^ | | | | | | |
| Any RTK^e^ | Yes | 16 (20) | 3 (8) | 7 (32) | 6 (26) | 0.055 |
|  | No | 66 (80) | 34 (92) | 15 (68) | 17 (74) |  |
| ERBB2 | Yes | 11 (13) | 1 (3) | 5 (23) | 5 (22) | 0.036 |
|  | No | 71 (87) | 36 (97) | 17 (77) | 18 (78) |  |
| PIK3CA | Yes | 15 (18) | 3 (8) | 6 (27) | 6 (26) | 0.096 |
|  | No | 67 (82) | 34 (92) | 16 (73) | 17 (74) |  |
| AKT1 | Yes | 26 (32) | 8 (22) | 10 (45) | 8 (35) | 0.153 |
|  | No | 56 (68) | 29 (78) | 12 (55) | 15 (65) |  |
| MTOR | Yes | 52 (63) | 21 (57) | 16 (73) | 15 (65) | 0.458 |
|  | No | 30 (37) | 16 (43) | 6 (27) | 8 (35) |  |
| KRAS | Yes | 13 (16) | 6 (16) | 4 (18) | 3 (13) | 0.892 |
|  | No | 69 (84) | 31 (84) | 18 (82) | 20 (87) |  |
| MEK1/2 (MAP2K1/2) | Yes | 20 (24) | 11 (30) | 6 (27) | 3 (13) | 0.320 |
|  | No | 62 (76) | 26 (70) | 16 (73) | 20 (87) |  |
| Cell cycle | | | | | | |
| CDK4/6 | Yes | 29 (35) | 15 (41) | 9 (41) | 5 (22) | 0.273 |
|  | No | 53 (65) | 22 (59) | 13 (59) | 18 (78) |  |
| Hedgehog | | | | | | |
| SMO | Yes | 6 (7) | 3 (8) | 1 (5) | 2 (9) | 0.840 |
|  | No | 76 (93) | 34 (92) | 21 (95) | 21 (91) |  |

^a^ The P value was calculated by the Chi-Square test.

^b^ The percentage was calculated in relation to all patients of the respective histological subtype (high-grade serous n=37, endometrioid n=22, clear cell n=23), if not otherwise indicated.

^c^ The percentage was calculated in relation to the overall cohort (n=82).

^d^ Among RTK, only ERBB2 had a postulated actionability in more than 5 patients, and was included in a separate analysis. No separate analysis was performed for MET, IGF1R, RET, EGFR, and KDR.

^e^ Indicates actionability for any of the analyzed RTK, namely ERBB2, MET, IGF1R, RET, EGFR, and KDR.

RTK, receptor tyrosine kinase
